# Japanese Encephalitis Virus Upregulates the Expression of SOCS3 in Mouse Brain and Raw264.7 Cells

**DOI:** 10.3390/v6114280

**Published:** 2014-11-10

**Authors:** Xiangmin Li, Qiaoyan Zhu, Qishu Cao, Huanchun Chen, Ping Qian

**Affiliations:** 1 State Key Laboratory of Agricultural Microbiology, Huazhong Agricultural University, Wuhan 430070, Hubei, China; E-Mails: lixiangmin@mail.hzau.edu.cn (X.L.); chenhch@mail.hzau.edu.cn (H.C.); 2 Laboratory of Animal Virology, College of Veterinary Medicine, Huazhong Agricultural University, Wuhan 430070, Hubei, China; E-Mails: qiaoyang306@126.com (Q.Z.); caoqishu19851127@163.com (Q.C.)

**Keywords:** Japanese encephalitis virus, SOCS3, gene expression profile, centralnervous system

## Abstract

Japanese encephalitis virus (JEV) is one of the pathogens that can invade the central nervous system, causing acute infection and inflammation of brain. SOCS3 protein plays a vital role in immune processes and inflammation of the central nervous system. In this study, Raw264.7 cells and suckling mice were infected with JEV, and SOCS3 expression was analyzed by the gene expression profile, semiquantitative RT-PCR, qRT-PCR, immunohistochemistry (IHC) and Western blot. Results indicated that 520 genes were found to be differentially expressed (fold change ≥ 2.0, *p* < 0.05) in total. The differentially regulated genes were involved in biological processes, such as stimulus response, biological regulation and immune system processes. JEV early infection could induce SOCS3 expression, upregulating both the mRNA and protein levels in Raw264.7 cells in a time-dependent manner. The SOCS3 expression was much lower in Raw264.7 cells infected with inactivated JEV than wild-type JEV. *In vivo*, SOCS3 protein was also found to upregulate the expression of mRNA and protein in JEV-infected mouse brain. Taken together, our data showed that JEV early infection could induce the upregulation of SOCS3 expression, both *in vitro* and *in vivo*, providing the basic theoretical foundation for future research on the invasion mechanism of JEV.

## 1. Introduction

Japanese encephalitis (JE) is a common disease in Southern and Eastern Asia, including Japan, India, China and South Korea. Japanese encephalitis virus (JEV), the etiological agent of JE, is a mosquito-borne flavivirus belonging to the family, *Flaviviridae*, which also contains yellow fever virus (YFV), dengue virus (DEV), West Nile virus (WNV), tick-borne encephalitis virus (TBEV) and St. Louis encephalitis virus (SLEV) [1]. The JEV genome is a single-stranded positive-sense RNA genome, roughly 11 kb in length, containing 5' and 3' non-translated regions (NTRs), three structural proteins (C, prM/M, E) and seven nonstructural proteins (NS1, NS2A, NS2B, NS3, NS4A, NS4B and NS5) [2]. JEV can cause an acute encephalitis and damage to the central nervous system (CNS). Approximately 30,000–50,000 JE cases and up to 10,000–15,000 deaths occur worldwide each year [3,4]. Among JE cases, approximately 25%–30% are fatal, and 50% result in permanent neuropsychiatric sequelae [3,5,6]. Despite the existence of a vaccine to prevent JEV, no specific and effective antiviral drug is available to treat JEV infection. Therefore, it is important to explore the invasion mechanism of JEV to develop antiviral drugs.

Most viruses encode proteins that inhibit the innate immune response to viral infection and promote viral replication [7,8]. Type I interferons (IFNs) function as the first line of defense against viral infections by modulating cell growth, establishing an antiviral state and influencing the activation of various immune cells. It is known that the Janus kinase signal transducer and activation of transcription (Jak-Stat) pathway can play a vital role in IFN response signaling [7]. DEV, which also belongs to the flavivirus family, cannot be inhibited by IFN in mice where the IFN-receptor is deficient [9] and in cultured cell systems; the mechanism of modulation was that IFN-α/β blocked the production of the negative-sense viral RNA [10]. Some scholars have confirmed that JEV infection can block the Jak-Stat signaling pathway induced by IFN-α [11]. It was further verified that the NS5 protein of JEV could inhibit the IFN-α-induced Jak-Stat signaling pathway through a mechanism mediated by a protein tyrosine phosphatase, which was identified as a negative regulator of the signaling pathway [12].

Suppressors of cytokine signaling (SOCS) proteins are intracellular, cytokine-inducible proteins that inhibit cytokine signaling in numerous cell types, including cells of the immune system and CNS [13]. The SOCS family is composed of eight members, and each has different physiological functions in various biological processes, but the well-described function is the negative regulation of the Jak-Stat signaling pathway [14,15,16,17]. SOCS1 and SOCS3 are expressed by immune cells and neurocytes and play vital roles under various neuroinflammatory or neuropathological conditions [13].

Under normal conditions, the SOCS3 mRNA level is low and often undetectable. However, SOCS3 expression is frequently increased by some cytokines and chemokines, and the mode of expression is dependent on the type of stimulating factors and cells [18]. In cells of the CNS and immune system, it has been reported that SOCS3 can be induced by stimuli, such as IL-6, IL-10, IFN-β and lipopolysaccharide (LPS) [13,19]. Recently, Kundu *et al*. reported that JEV could induce the expression of SOCS1 and SOCS3 in macrophages [20]. However, it is still unclear what are the roles of SOCS3 protein under the JEV infection condition.

In this study, we detected the *in vivo* and *in vitro* expression of SOCS3 following JEV infection with the purpose of providing a basic theoretical foundation for future research on JEV neuroinflammation.

## 2. Materials and Methods

### 2.1. Virus and Antibodies

The JEV strain used in this study was the SX09S01 strain isolated from infected swine brain by our laboratory [21] and propagated into BHK-21 cells. Viral titers were determined for BHK-21 cells by plaque assay and directly used in this study. JEV was inactivated for one hour in a water bath at 56 °C, and its activation was checked by culturing the virus in BHK-21 cells for three passages.

Rabbit polyclonal anti-SOCS3 antibody was purchased from Santa Cruz Biotechnology (Paso Robles, CA, USA). Mouse monoclonal anti-actin antibody was purchased from Sigma (Shanghai, China). The monoclonal antibody for the JEV E protein was a gift from Dr. Cao in our laboratory.

### 2.2. Cell Lines and Mice

BHK-21 and Raw264.7 cells (a murine macrophage-like cell line) were cultured in DMEM supplemented with 10% FBS and incubated at 37 °C, 5% CO_2_ in a humidified incubator.

In this study, Kunming suckling mice were used. Mice were purchased from the Center for Animal Disease Control, Hubei Province, and all experiments were conducted with approval of the Animal Committee of Huazhong Agricultural University.

### 2.3. Virus Infection

Monolayers of Raw264.7 cells cultured in 12-well plates were initially adsorbed with virus at 5 MOI (multiplicity of infection) for 1 h at 37 °C. Unbound virus was removed from the cells by gentle washing with PBS buffer, and the cells were incubated with fresh DMEM containing 10% FBS and 1% penicillin-streptomycin solution. At different time points after infection, cells were harvested directly for RNA extraction or protein analysis.

Kunming mice of 3 days were subcutaneously injected with JEV (SX09S01) using a viral titer of 1.58 × 10^5^ tissue culture infective dose (TCID)_50_, and the brains were collected at different time points post infection (p.i.). The brain tissues were combined with 1 mL cold PBS buffer, and total RNA and protein were extracted.

### 2.4. Gene Chip and Bioinformatics

The Raw264.7 cells infected with JEV for 2 h were directly treated with TRIZOL^®^ Reagent (Invitrogen, Grand Island, NY, USA) and sent to the KangChen Bio-tech Corporation (Shanghai, China) for analysis using microarrays. Oligo probes of 41,000 mouse genes were used with 4 × 44 K Agilent technology, and the experiments were performed in Cy3 and Cy5 independent changing labels for hybridizations. Data extraction from images was done by using Agilent Feature Extraction software. Hierarchical cluster, gene ontology and pathway analysis were analyzed by using SAS (ShanghaiBio Analysis System, Shanghai, China).

### 2.5. RNA Extraction, Semiquantitative RT-PCR and QRT-PCR

The total RNAs from mouse brain and Raw264.7 cells were isolated using the TRIZOL^®^ Reagent (Invitrogen, Grand Island, NY, USA) according to the manufacturer’s protocol. For cDNA synthesis, 1 μg of RNA was used as a template using 100U of avian myeloblastosis virus (AMV) reverse transcriptase (Toyobo, Osaka, Japan), 10 mM dNTPs and 10 μM primers. The reaction conditions were 42 °C for 25 min and 99 °C for 5 min. The semiquantitative and qRT-PCR were performed using the primers indicated in Table 1.

**Table 1:** Primers used in this study.

Primer Name	Primer Sequence (5'-3')F= Forward, R= Reverse
SOCS3	F=5'-GAGCGGATTCTACTGGAGCG-3'
	R=5'-TGGATGCGTAGGTTCTTGGTC-3'
GAPDH	F=5'-GCCCAAGATGCCCTTCAGT-3'
	R=5'-CCTTCCGTGTTCCTACCCC-3'

The master mix for PCR reaction contained: 500 ng of cDNA, 10 mM dNTPs, 10 μM primers and 2.5 U rTaq. The mixture was heated at 94 °C for 4 min and 30 cycles of 94 °C for 30 s, 60 °C for 30 s and 72 °C for 30 s and, then, a single 10 min extension at 72 °C. Relative quantification of the genes SOCS3 and GAPDH were measured by qRT-PCR using a Roche Light Cycler 480. To analyze SOCS3 expression, the expression of the housekeeping gene, GAPDH, was determined. For PCR, the Q-PCR SYBR Green Mastermix (Toyobo, Osaka, Japan) was used, and the gene fragment was amplified for 45 cycles. The master mix for PCR reaction contained: 500 ng of cDNA, 1 μL each of the primers and SYBR green mixture (25 μL of total volume). The PCR products were detected by agarose gel electrophoresis under UV light.

### 2.6. Western Blot Analysis

Mouse brain tissue or Raw264.7 cells were lysed with NP40 lysis buffer containing protease inhibitor on ice for at least 1 h. The concentration of total protein was determined using the BCA protocol following the manufacturer’s instructions. Equal concentrations of protein lysates were then combined with sodium dodecyl sulfate (SDS) sample loading buffer (50 mM Tris-HCl (pH6.8), 2% SDS, 10% glycerol, 0.1% bromophenol blue, 0.5% β-Mercaptoethanol) and treated at 95 °C for 10 min. The proteins were then separated by SDS-PAGE and transferred to PVDF membranes. The membranes were blocked with 10% skim milk and 1% BSA in TBS-T for 2 h at room temperature. To detect SOCS3 protein expression, blots were probed overnight with a 1:300 dilution of anti-SOCS3 Ab at 4 °C and then incubated with a 1:5000 dilution of HRP-conjugated anti-rabbit immunoglobulin G (IgG) for 1 h at room temperature. Finally, the SOCS3 protein was detected using an ECL system. The membrane was stripped and then re-probed with β-actin using the same method.

### 2.7. Histological Analysis

Whole brains were fixed in a 10% formaldehyde solution for 3 days, embedded in paraffin, serially sectioned and then stained with hematoxylin and eosin. To detect the expression of JEV E and SOCS3 protein, immunohistochemistry was used. In this process, slides were incubated with a 1:100 dilution of anti-E or SOCS3 Ab overnight at 4 °C and then treated with a 1:5000 dilution of HRP-conjugated anti-rabbit immunoglobulin G (IgG) for 1 h at room temperature. Finally, the IHC slides were observed under the microscope.

### 2.8. Statistical Analysis

To ascertain changes in the expression of the genes, the differences between the expression of SOCS3 and GAPDH were calculated using the 2^−∆∆CT^ method [22]. The results of quantitative RT-PCR were presented as x ± SD (*n* = 4), where x was the mean of the data of the four replicates. All data were analyzed using GraphPad Prism 5 software. Using the unpaired Student’s *t*-test, the differences were analyzed and the *p*-value calculated.

## 3. Results

### 3.1. Gene Expression Profiles in Raw264.7 Cells Infected with JEV

**Figure 1:**
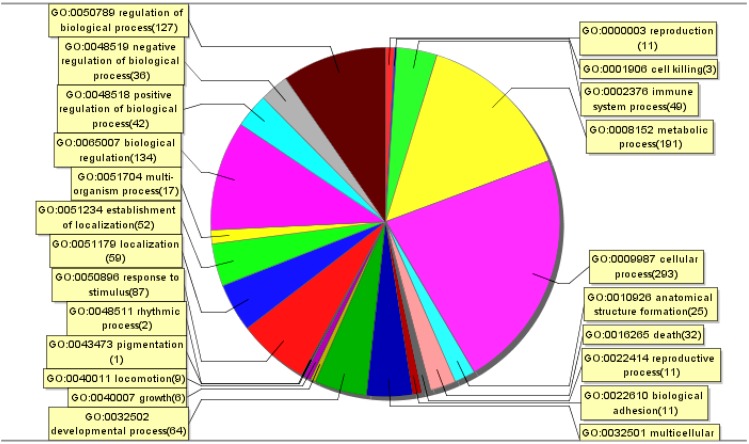
Enriched Gene Ontology terms in the biological process category among differentially expressed genes. After the mRNA microarray assay, significantly enriched Gene Ontology analysis in the biological process category among differentially expressed genes (fold change over non-infected ≥2) in Raw264.7 cells infected with SX09S-01 at 5 MOI for 2 h was performed by using SAS (ShanghaiBio Analysis System). Each color section represents a different biological process, and the gene number enriched in this section was shown following the biological process name.

A whole genome array was used to perform a systematic analysis of the mRNA expression profile of Raw264.7 cells infected with the SX09S-01 strain at 5 MOI for 2 h. The genes identified with significantly differential expression usually demonstrated a ≥2-fold change. Approximately 14,450 genes were detected on the chip, of which 520 genes were differentially expressed (fold change ≥ 2.0, *p* < 0.05). We performed a functional classification of mRNA transcripts and pathway analysis to elucidate the correlation between the gene expression pattern and JEV infection-induced biological processes. We found that these genes were involved in biological processes, such as cellular processes, stimulus response, biological regulation and immune system processes (Figure 1).

Additionally, the significant pathways of differentially expressed genes in the cells were known to be involved in metabolic pathways, the cell cycle, cytokine-cytokine receptor interactions and others (Table 2).

Compared to the mock-treated cells, 272 genes in the JEV-infected cells were significantly downregulated and 248 genes were upregulated. Among the significantly upregulated genes, we found several genes of interest, such as Il-10, Jak2, Ccl2, SOCS3, Stat1, Stat3, Il-15, and Stat2. Based on these preliminary results, we focused on the SOCS3 gene to determine how JEV upregulates its expression in Raw264.7 cells and suckling mice.

**Table 2:** Significant pathway of the differentially expressed genes in Japanese encephalitis virus (JEV)-infected Raw264.7 cells.

Pathway Name	No. of Genes	*p*-Value
IFN alpha signaling pathway	2	0.0026
IFN gamma signaling pathway	2	0.0014
IL-10 Anti-inflammatory signaling pathway	2	0.0026
p38 MAPK signaling pathway	3	0.0018
Cell cycle	14	0.0
Chemokine signaling pathway	9	0.0
Cytokine-cytokine receptor interaction	13	0.0
Cytosolic DNA-sensing pathway	10	0.0
JAK-STAT signaling pathway	6	0.0012
Metabolic pathways	41	0.0
NOD-like receptor signaling pathway	6	0.0
p53 signaling pathway	7	0.0
RIG-I-like receptor signaling pathway	5	2.0 × 10^−4^
Toll-like receptor signaling pathway	7	0.0

### 3.2. Change of SOCS3Expression in Raw264.7 Cells Infected with JEV

**Figure 2:**
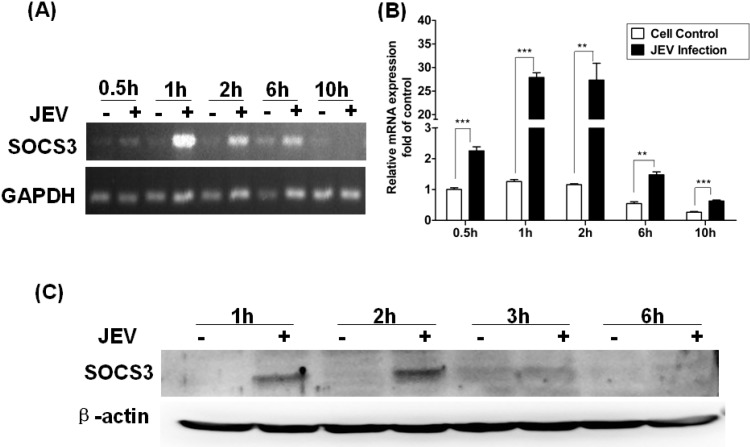
Changes in the expression of SOCS3 at the mRNA and protein levels in Raw264.7 cells infected with JEV. Raw264.7 cells were infected with JEV at 5 MOI, and samples were collected at different time points post infection (p.i.). Semiquantitative (**A**) and quantitative (**B**) RT-PCR was performed with RNA extracted from mock-infected or JEV-infected RAW264.7 cells. Results of quantitative RT-PCR were expressed as the fold change in relative SOCS3 mRNA expression over the control. Data are expressed as the mean ± SD from four independent experiments. (**C**) Expression of SOCS3 protein in mock-infected and JEV-infected RAW264.7 cells was analyzed by Western blotting. Differences were assessed by using unpaired Student’s *t*-tests. ** *p* < 0.01, *** *p* < 0.005.

To further confirm the expression of SOCS3 induced by JEV, we analyzed levels of mRNA and protein of SOCS3 at different time points post infection (p.i.) by semiquantitative RT-PCR and qRT-PCR. The results of semiquantitative RT-PCR showed that SOCS3 mRNA expression was slightly induced at 30 min p.i., peaked at 1–2 hand was visibly diminished at 6–10 h (Figure 2A).The same results were determined by qRT-PCR. The expression of SOCS3 was upregulated at 30 min after JEV infection. The expression peaked at 1 h and 2 h p.i. (Figure 2B), and the fold changes were 22.4 ± 3.55 and 23.5 ± 4.22, respectively. A similar result was obtained by Western blotting. The SOCS3 protein was expressed at 1 h and 2 h p.i. in infected cells, but not in control cells (Figure 2C). These results were in agreement with semiquantitative and qRT-PCR. These data confirmed that SOCS3 expression was significantly upregulated in JEV-infected Raw264.7 cells at an early stage and appeared to be time dependent.

### 3.3. Level of Expression of SOCS3 in Raw264.7 Cells Infected with Inactivated JEV

**Figure 3:**
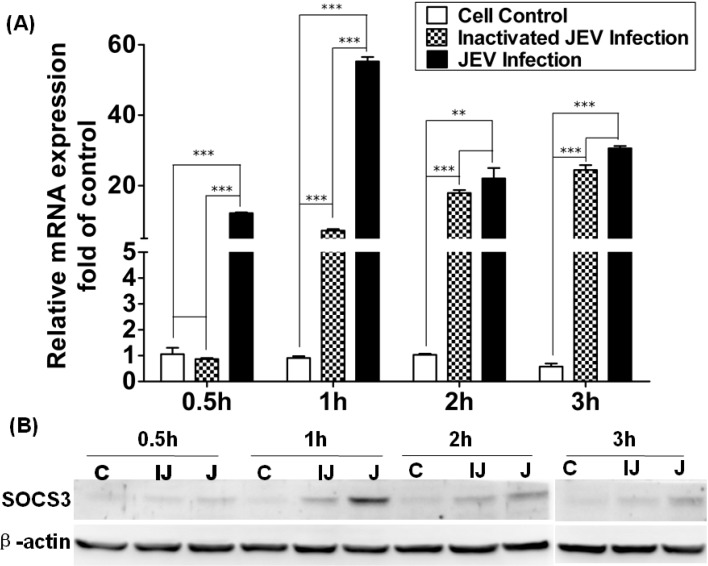
Expression of SOCS3 in Raw264.7 cells infected with wild-type or inactivated JEV. (**A**) Quantitative RT-PCR was performed with RNA extracted from mock-infected, inactivated JEV or wild-type JEV-infected RAW264.7 cells after different time points, post-infection. Results were expressed as the fold change in relative mRNA expression over the control. (**B**) Expression of SOCS3 protein in mock-infected (C), inactivated (IJ) or wild-type JEV-infected (J) RAW264.7 cells was analyzed by Western blotting. Data are expressed as the mean ± SD from four independent experiments, and significance was evaluated by GraphPad Prism 5 software. Differences were assessed by using unpaired Student’s *t*-tests. ** *p* < 0.01, *** *p* < 0.005.

To investigate the mechanism for how to induce SOCS3 expression, Raw264.7 cells were infected with wild-type and inactivated JEV, and then, SOCS3 mRNA and protein expression was detected by qRT-PCR and Western blotting. The results showed that the ability of inactivated JEV to induce SOCS3 was observably lower than that of wild-type JEV, and there appeared to be a significant difference in induction between inactivated JEV and wild-type JEV at 1 h (Figure 3A). As shown in Figure 3B, the inactivated JEV could induce the expression of SOCS3 protein at 2 h and 3 h p.i. Compared with cells infected with JEV, the level of expression of SOCS3 proteins induced by inactivated JEV were low at different time points p.i., and wild-type JEV could induce SOCS3 protein expression the same as in Figure 2.

### 3.4. Expression of SOCS3 in Mouse Brain Infected with JEV

**Figure 4:**
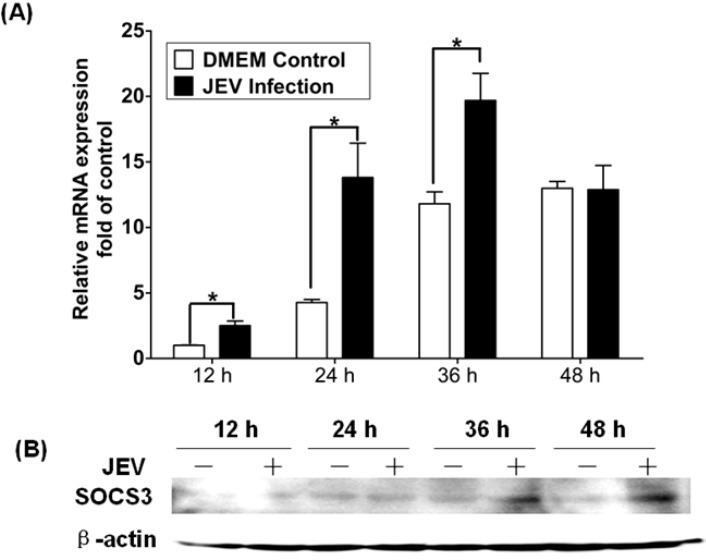
Change of SOCS3 expression in mouse brain infected with JEV. Mice brains were subcutaneously injected with JEV (SX09S01) using a viral titer of 1.58 × 10^5^ tissue culture infective dose (TCID)_50_, and samples were collected at different time points post-infection. (**A**) Quantitative RT-PCR was performed with RNA extracted from mock-infected or wild-type JEV-infected mice brains 12 h, 24 h, 36 h and 48 h p.i. The results were expressed as the fold change in relative mRNA expression over the control. (**B**) Expression of SOCS3 protein in mock-infected or wild-type JEV-infected mice brains was analyzed by Western blotting. Data are expressed as the mean ± SD from four independent experiments, and significance was evaluated by GraphPad Prism 5 software. Differences were assessed by using unpaired Student’s *t*-tests. * *p* < 0.05.

To illuminate the relationship between the expression of SOCS3 and encephalitis *in vivo*, suckling mice were subcutaneously injected with JEV. *In vivo*, it will take 12 h for JEV to reach the three-day mice brain by the subcutaneously route, and significant clinical signs will appear 36 h p.i. Therefore, the brains were collected at 12-, 24-, 36- and 48-h time points after infection. Using qRT-PCR, we found that levels of SOCS3 mRNA were upregulated at 12 h, peaked at 24 h and returned to normal levels at 48 h (Figure 4A). JEV-specific induction of SOCS3 mRNA in the brain would be ~3-fold at 24 h and less than 2-fold at 36 h. The increased levels of SOCS3 mRNA in DMEM-treated animals could be due to the treatment itself (injection of DMEM). However, for all of the tested time points, the upregulation of SOCS3 in infected brains was significantly higher than the treatment alone. The SOCS3 protein bands were examined at 36 h and 48 h post-JEV infection using Western blotting (Figure 4B). Protein expression was consistent with those for mRNA expression.

### 3.5. Histological Analysis and IHC Detection of Mouse Brain Infected with JEV

**Figure 5:**
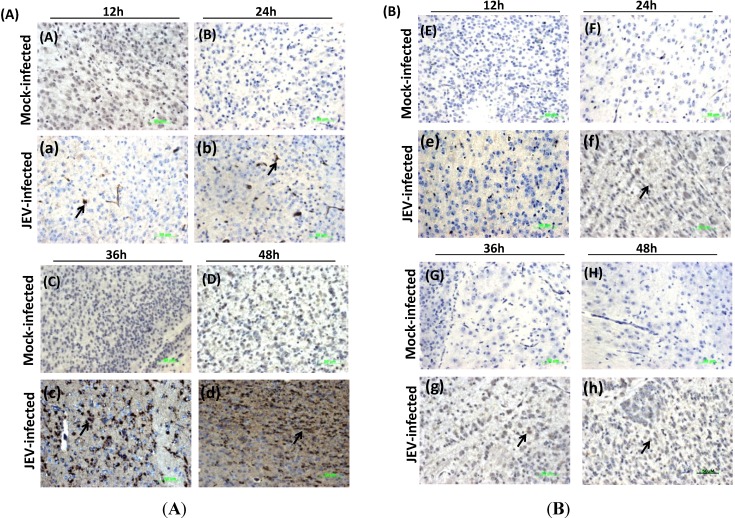
Immunohistochemistry analysis for JEV envelope (E) protein (**A**) or SOCS3 (**B**) expression in mock-infected or infected mice brains at different time points post-infection. The same mice brains from Figure 4 were fixed in a 10% formaldehyde solution for three days and embedded in paraffin, serially sectioned and then stained with monoclonal antibody against JEV E or SOCS3. The positive signals are shown by the arrow. (**A**): (A–D): mock-infected mice brains; (a–d): JEV-infected mice brains; slides were stained with monoclonal antibody against JEV E. (**B**): (E–H): mock-infected mice brains; (e–h): JEV-infected mice brains. Slides were strained with monoclonal antibody against SOCS3. Magnification = 40×; scale bar = 50 μM.

To verify the expression of the SOCS3 protein in mice brain infected with JEV, we performed the relational experiment of H&E and IHC. Using H&E staining, a series of pathological phenomenon were identified in the JEV-infected brains, including edema fluid infiltration, perivascular inflammatory cell invasion, proliferation of glial cells, necrosis in the central chromatolytic neurons and eosinophilic phenomenon. We also confirmed that mice brain infected with JEV showed the brown positive signal for the JEV antigen in JEV-infected brains, not appearing in the DMEM control group (Figure 5). We could detect the brown positive signals of the SOCS3 antigen in the JEV-infected brain at different time points (Figure 5). It was further confirmed that SOCS3 proteins were induced in mice brain by JEV and are in accordance with the expression of SOCS3 and encephalitis *in vivo* (Figure 4).

## 4. Discussion

Central nervous system (CNS) diseases are a vital issue of global public health and safety. JEV, which is one of the pathogens that can cause CNS infections, can invade the brain tissue and cause brain inflammation and damage to the CNS. It has been reported that the SOCS proteins, especially the SOCS3 protein, play a vital role in immune processes and inflammation [13,23].

As previously reported, a number of different viruses can induce SOCS3 expression, including Coxsackie virus [24], HIV-1 [16], HCV [25], HBV [26], HSV-1 [27,28], RSV [29,30] and influenza A virus [31,32]. Expression of SOCS3 depends on the different virus, such that influenza A virus can induce a two- to nine-fold expression of SOCS3; HCV core protein only induces approximately 2.5-fold levels of SOCS3 protein expression. In this study, we preliminarily demonstrated that JEV could upregulate SOCS3 expression in Raw264.7 cells according to gene expression profile analysis. Through Gene Ontology (GO) and pathway analysis, JEV could cause differentially expressed changes in genes involved in the biological process of cytokine-cytokine receptor interactions and pathways involving receptor interactions (Figure 1 and Table 2).

Among these different genes, we focus on the SOCS3 gene, which negatively regulates the Jak-Stat signaling pathway. It has been indicated that SOCS1 and SOCS3 proteins are associated with neuroinflammation. It was also confirmed that expression of the SOCS3 gene could be induced in mouse brain by gene expression profiling [6]. To further clarify the expression of SOCS3, the mRNA and protein levels of SOCS3 were detected in Raw264.7 cells. JEV can induce the expression of SOCS3, and that expression was time dependent and independent viral replication (Figure 2). Yokota *et al*. have shown that HSV-induced SOCS3 expression is dose-dependent in FL cell [30]. Other reports have shown that the LPS-induced upregulation of SOCS3 expression of mRNA and protein is time dependent in murine macrophage and microglial cell lines and in primary microglia, and RSV-induced SOCS3 expression is also time dependent in epithelial cells [19,30].

Hashimoto *et al*. have confirmed that inactivated RSV could not induce a significant change in the mRNA of SOCS3 in HEp-2 cells, and their results implied that SOCS3 expression required viral gene expression [30]. The HCV core protein can induce SOCS3 expression, and the viral 5'-tirphosphate RNA of influenza A can also increase SOCS3 expression and reduce STAT1 phosphorylation [25,31]. In this study, inactivated JEV could also induce low levels of SOCS3 expression at the mRNA or protein level compared with the control cells or JEV-infected cells (Figure 3). This maybe due to some cytokines that present in the virus sample, because the virus used in this study was not purified. These preliminarily results implied that viral proteins may be required to induce the upregulation of SOCS3 expression at early stages of infection. It is under investigation how JEV upregulated the expression of SOCS3 protein.

WNV and TBEV both belong to the flavivirus family and can induce the upregulation of SOCS1 and SOCS3 mRNA in mouse brain [33]. Yang *et al*. had clarified the upregulated expression of SOCS3 in the brains of JEV-infected mice through gene chip analysis [6]. To study whether JEV can induce the expression of SOCS3 in mouse brain and the relationship between SOCS3 protein and inflammation in CNS, suckling mice were subcutaneously injected with JEV. JEV can cross the blood brain barrier and invade the brain and cause a series of histopathological changes, including neuronal necrosis, proliferation of glial cells and eosinophilic phenomenon. Baker *et al*. have shown that SOCS1 and SOCS3 proteins play critical roles in the control of CNS immunity [13]. Here, we also showed that JEV could upregulate the expression of SOCS3 in mice brain (Figure 4 and Figure 5). These results indicated that the inflammation of brain caused by JEV might be related to the upregulation of SOCS3 protein. Recently, Kundu *et al*. reported that JEV can modulate the expression of SOCS in macrophages, and it may implicate the hosts’ innate immune response. In our laboratory, we also find that JEV infection could induce the expression of p-STAT2/STAT2 or p-NF-κB proteins and activate the IFN signaling pathway. This result provides an insight into the role of JEV infection in modulating the Jak-Stat pathway with the help of SOCS, leading to the generation of an antiviral innate immune response.

In conclusion, we found the upregulation of SOCS3 gene in Raw264.7 cells by gene expression profile analysis and further confirmed that JEV could induce the SOCS3 expression *in vivo* and *in vitro*. Upregulation of SOCS3 protein may play a vital role in the inflammation of brain induced by JEV.
